# Negative regulation of initial steps in skeletal myogenesis by mTOR and other kinases

**DOI:** 10.1038/srep20376

**Published:** 2016-02-05

**Authors:** Raphael A. Wilson, Jing Liu, Lin Xu, James Annis, Sara Helmig, Gregory Moore, Casey Timmerman, Carla Grandori, Yanbin Zheng, Stephen X. Skapek

**Affiliations:** 1 Division of Hematology/Oncology, Department of Pediatrics, University of Texas Southwestern Medical Center, Dallas, TX, USA.; 2 Quellos High Throughput Screening Core, Department of Pharmacology, Institute for Stem Cell and Regenerative Medicine, University of Washington, Seattle, WA, USA.

## Abstract

The transition from a committed progenitor cell to one that is actively differentiating represents a process that is fundamentally important in skeletal myogenesis. Although the expression and functional activation of myogenic regulatory transcription factors (MRFs) are well known to govern lineage commitment and differentiation, exactly how the first steps in differentiation are suppressed in a proliferating myoblast is much less clear. We used cultured mammalian myoblasts and an RNA interference library targeting 571 kinases to identify those that may repress muscle differentiation in proliferating myoblasts in the presence or absence of a sensitizing agent directed toward CDK4/6, a kinase previously established to impede muscle gene expression. We identified 55 kinases whose knockdown promoted myoblast differentiation, either independently or in conjunction with the sensitizer. A number of the hit kinases could be connected to known MRFs, directly or through one interaction node. Focusing on one hit, *Mtor*, we validated its role to impede differentiation in proliferating myoblasts and carried out mechanistic studies to show that it acts, in part, by a rapamycin-sensitive complex that involves Raptor. Our findings inform our understanding of kinases that can block the transition from lineage commitment to a differentiating state in myoblasts and offer a useful resource for others studying myogenic differentiation.

The transition between a proliferating, committed progenitor cell and one that is actively differentiating represents a fundamental step in cellular and, ultimately, organism development. Skeletal muscle formation represents a particularly important example as a process choreographed by a limited number of transcription factors determining muscle lineage and driving the differentiation program^1^,^2^. Elegant mouse studies show that the lineage is determined by expression of MyoD or Myf5, two members in a family of basic helix-loop-helix (bHLH) transcription factors^3^,^4^. Certain extracellular signals guide early stages of lineage commitment: surface ectoderm-derived Wnts and Shh from the floor plate and notochord drive the initial expression of Myf5 and MyoD in the dermomyotome, whereas Notch- and BMP-derived signals repress their expression^5^,^6^,^7^,^8^. However, the mere expression of myogenic bHLH proteins is not sufficient to start the differentiation cascade in committed myoblasts. Hence, a major question remains: How is the differentiation program suppressed in proliferating myoblasts?

The function of myogenic bHLH proteins, which form heterodimers with *E2a* gene products to bind to “E-boxes” in regulatory elements of muscle-specific genes^1^, hinges largely on physical and functional interactions with a variety of cofactors including other sequence-specific transcriptional activators like Mef2 proteins^9^,^10^; cell cycle proteins including the retinoblastoma tumor suppressor, Rb^11^,^12^; and histone and chromatin remodeling proteins p300 and PCAF^13^,^14^. In many cases, these interactions are controlled by kinases. For example, consistent with the fact that functional Rb is critical for myogenic bHLH protein activity^11^,^15^, excess Cyclin D1-associated Cyclin-dependent kinase (Cdk) 4 or 6 can impede muscle differentiation^16^, and expression of a Cdk inhibitor^16^ or chemical inhibition of Cdk4/6 activity^17^ enhances muscle gene expression. Kinases also positively influence the activity of MyoD- and Mef2-related proteins. Activation of p38 Mapk provides the clearest example, as its inhibition or forced activation in cultured myoblasts impedes or augments the expression of differentiation markers, respectively^18^,^19^.

Regulation of the myoblast-to-myocyte transition holds substantial importance for muscle development and disease. Major muscle groups representing 30–40% of the adult human body mass are defined during embryonic and perinatal development, yet adult skeletal muscle retains a capacity to increase muscle mass by hyperplastic and hypertrophic processes^6^,^20^. Similarly, muscle mass diminishes in physiological aging and a variety of diseases, and this is linked to loss of myonuclei and decreased synthesis of skeletal muscle proteins^6^,^21^,^22^. In another example, rhabdomyosarcoma represents a malignant soft tissue sarcoma composed of skeletal myoblast-like cells^2^,^23^. Although rhabdomyosarcoma expresses lineage-defining muscle proteins, the cells have lost the capacity to undergo terminal muscle differentiation^24^. This disease, in particular, may be viewed as an extreme example of derailed skeletal muscle differentiation.

To better understand the breadth of kinases that can block the differentiation program in proliferating myoblasts, we leveraged a well-characterized mammalian cell culture model in a high-content, cell-based screen. In contrast to other myogenic screens focusing on enhancers of muscle differentiation^25^,^26^ or muscle lineage specification^27^, we searched for kinases that block muscle gene expression in proliferating myoblasts. In this report, we describe our high-content screen and initial findings, bioinformatics analyses highlighting potential interactions between hit kinases and MRFs, and functional validation and mechanistic studies of mTOR, one of the hits.

## Results

### High content, cell-based screen to detect Myogenin induction in myoblasts

**Figure 1:**
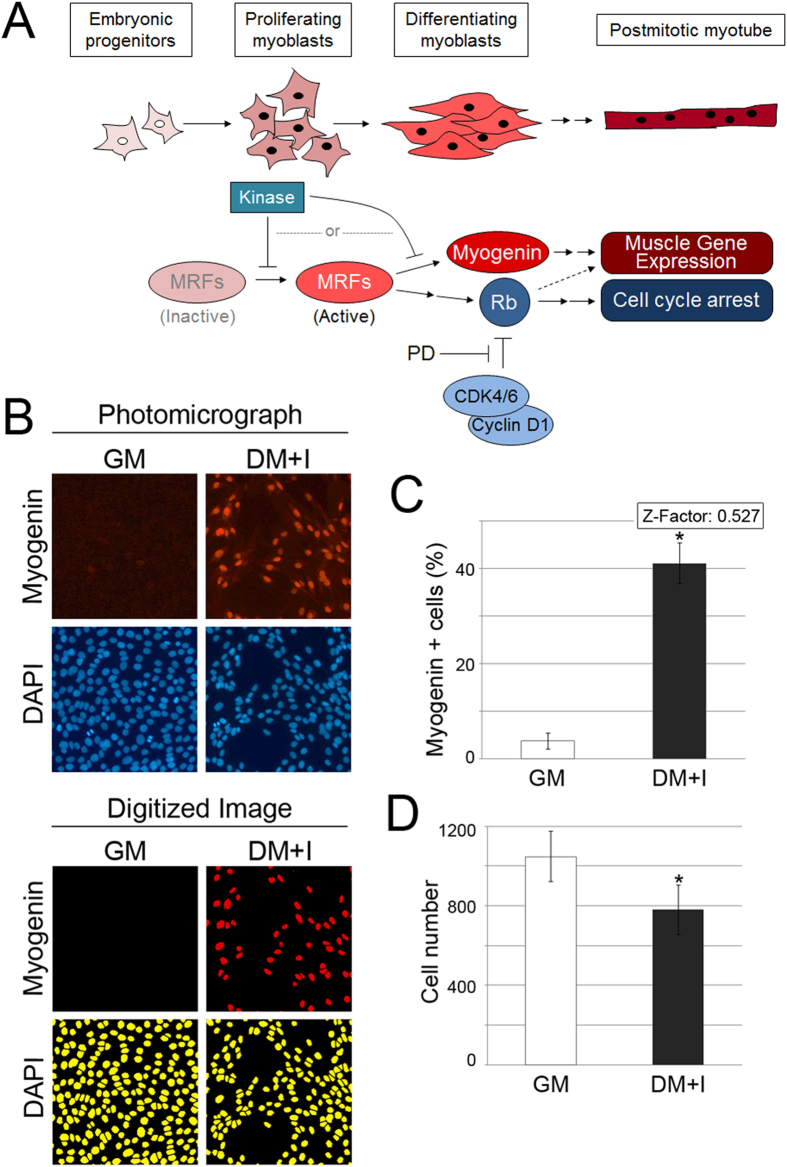
High content siRNA screen for kinases negatively regulating myogenic differentiation. (**A**) Schematic diagram showing morphological changes and important molecular events during skeletal myogenesis. (**B**) Immunofluorescence staining for Myogenin and DAPI in C2C12 cells grown in GM or DM + I (top) and digitized versions of images (bottom), used by MetaXpress software to count the nuclei. (**C,D**) Quantification of Myogenin-positive cells (**C**) producing a Z-factor of 0.527, and total cell number (**D**) in aggregate wells from 384-well plate from negative (GM) and positive (DM + I) controls. *p < 0.05 in GM vs. DM + I; error bars represent standard deviation.

We designed a high throughput screen to uncover kinases that block muscle gene expression in committed, but undifferentiated, myoblasts. Specifically, we leveraged an siRNA library targeting much of the mouse kinome to test how knockdown of individual kinases influenced the expression of Myogenin, a myogenic bHLH protein induced by MyoD binding to the *Myog* promoter^28^. We utilized the well characterized, mouse C2C12 myoblast model, which has been used for many years to illuminate fundamental steps in myogenesis. “Hits” represented those kinases for which knockdown increased the fraction of cells expressing Myogenin, even though the cells were maintained in mitogen-rich growth medium (GM) (Fig. 1A). Myogenin expression was assessed by fluorescence staining and quantified using automated fluorescence microscopy in cells cultivated in GM versus serum-poor differentiation medium with insulin (DM + I)(Fig. 1B). We established a robust assay window based on both Myogenin expression and decreased cell number due to the cell cycle arrest that normally accompanies differentiation (Fig. 1C,D). Recognizing the capacity for Cyclin D-Cdk4/6 to promote proliferation and blunt differentiation, we included an experimental arm with cells sensitized using PD 0332991 (Fig. 1A). This Cdk4/6 inhibitor blocks proliferation in these myoblasts and slightly increases Myogenin^17^; however, that small increase was not detectable in this screen (see below).

**Figure 2:**
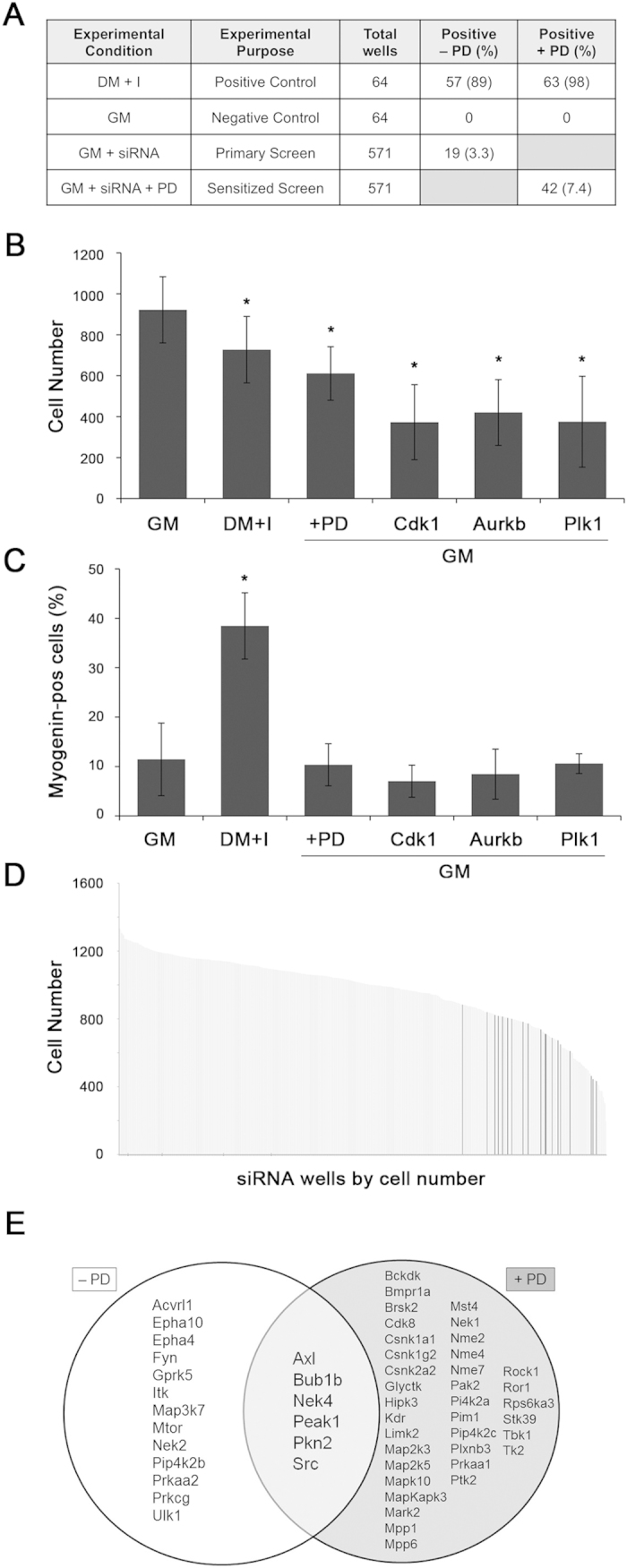
High content siRNA screen identifies a subset of kinases that block myogenin induction in myoblasts. (**A**) Chart showing experimental conditions and controls used in the screen and quantitation of hits under different conditions. (**B,C**) Charts showing total number of cells per well (**B**) or percent of cells expressing Myogenin (**C**) when cultivated in the indicated medium with or without PD 0332991 (PD) or or with siRNAs targeting individual kinases. (**D**) Chart showing average total cell number in experimental wells targeting each kinase. Gray and black bars signifying kinases scoring as negative or positive, respectively, for Myogenin induction. (**E**) Venn diagram showing the positively scoring “hits” with and without PD. PD, PD 0332991; GM, growth media; DM + I, differentiation media plus insulin; *p < 0.05; error bars represent standard deviation.

In the screen, an individual well scored positively if the knockdown both a) significantly increased the average fraction of Myogenin-positive cells in the four sites automatically sampled by the immunofluorescence microscope, and b) decreased the average number of cells in the triplicate wells to a value that is within one median absolute deviation (MAD) of the cell number for the DM control. In both cases, comparison was made to cells cultivated in GM and transfected with control siRNA. For cells receiving PD 0332991 as a sensitizer, total cell number was not included as a variable because this drug decreases myoblast cell number independently of siRNA knockdown^17^. “Hits” were called if two of the replicates (or all three when the sensitizer was included) were positive. To test these definitions, we applied them to the 64 negative and positive control wells containing myoblasts cultivated in GM or DM + I, respectively. None of the negative control wells scored positively whereas 89% and 98% of the positive control wells scored as such with either vehicle or PD 0332991, respectively (Fig. 2A).

Skeletal muscle differentiation is intimately coupled to arrest in G_0_/G_1_ phase of the cell cycle^2^. We discovered, though, that decreased total cell number was not sufficient for an individual kinase to score positively. As indicated above, none of the wells scored positively when cells were in GM plus PD 0332991, even though cell number significantly decreased (Fig. 2B,C). Our panel included well-characterized cell cycle regulators like *Cdk1*, *Aurkb*, and *Plk1*, the knockdown of which decreased cell number without increasing Myogenin (Fig. 2B,C). Indeed, focusing on the non-sensitized screen, the total cell number decreased by at least one median absolute deviation from the GM control in 138 wells, but only a small number of these displayed significantly increased Myogenin (Fig. 2D). Hence, merely arresting cell proliferation was not sufficient to initiate the muscle differentiation program in MyoD-expressing myoblasts.

### Kinases identified as negative regulators of muscle gene expression in proliferating myoblasts

The siRNA screen included triplicate plates containing 571 experimental wells, each of which targeted a single mouse kinase. We observed that knockdown of 19 of those kinases increased the Myogenin-expressing fraction and decreased the total cell number, providing a hit-rate of 3.3% for siRNA alone and 7.4% for siRNA plus the Cdk4/6 inhibitor (Fig. 2A and Supplementary Figure S1). The magnitude of the effect ranged from a Z-score of 0.9 (*Fyn*) to 3.5 (*Nek2*) without the Cdk4/6 inhibitor and from 1.5 (*Brsk2*) to 4.8 (*Mapkapk3*) with the sensitizer (Supplementary Table S1). Six of the 19 hits without the sensitizer also scored positively when the sensitizer was included (Fig. 2E). Taking into account those “double hits” (*Axl*, *Bub1b*, *Nek4*, *Peak1*, *Pkn2*, and *Src*), 55 (9.6%) of the kinases scored positively.

**Figure 3:**
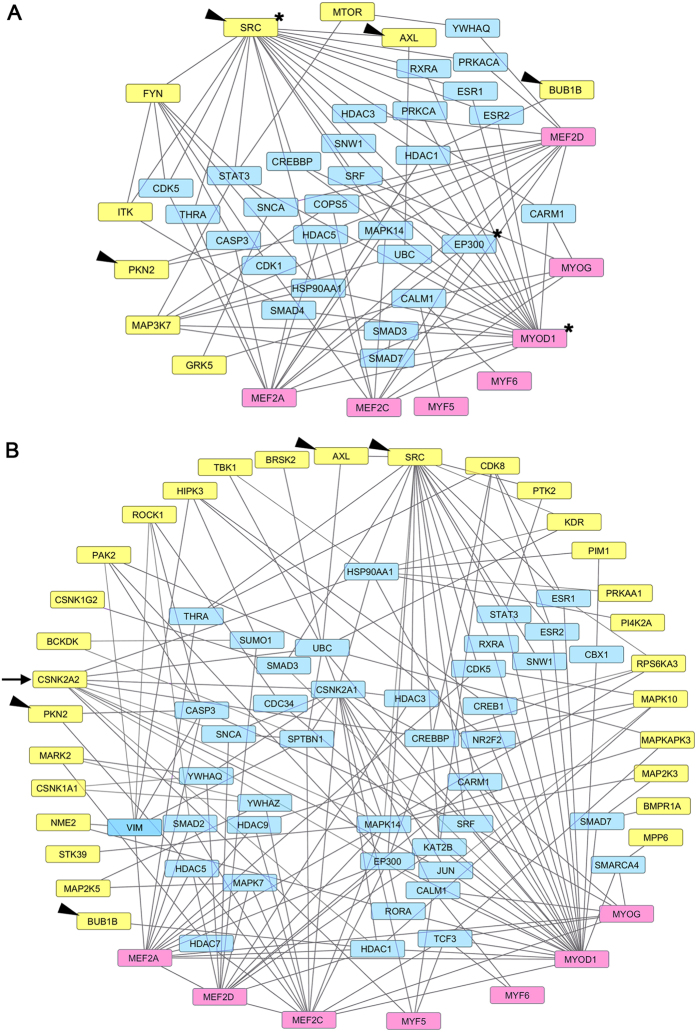
Hit kinases intersect with individual proteins in biological pathways that are relevant to skeletal muscle differentiation. Genetic interaction network for hits without PD (**A**) and with PD (**B**) linking MyoD- and Mef2-family MRFs (pink) to selected kinases (yellow). Note that one hit kinase, CSNK2A2 (**B**, arrow), shows a direct connection to MYF5, while others represent all hit kinases interacting semi-directly through one interaction node (light blue). Asterisks (**A**) denote the hit kinase, MRF, and interacting node with the greatest number of interactions. Several of these semi-direct regulators were identified in both the sensitized screen and non-sensitized screens (arrowheads).

We studied how hit kinases were related to each other and to MyoD- and Mef2-family myogenic regulatory factors (MRFs) by exploring known human genetic interaction networks using the HPRD and BioGRID databases (Fig. 3A,B). First, we observed that only one kinase, *Csnk2a2*, which scored positively with PD 0332991, had a direct connection with one of the myogenic regulators, *Myf5* (Fig. 3B). Second, nine of the 19 hits from the primary siRNA screen (and 28 from the sensitized screen) were just one interaction node away from one of the MRFs; we considered them “semi-direct” regulators. Four of these semi-direct regulators – *Src*, *Axl*, *Bub1b*, and *Pkn2* – were identified in both screens. *Src* interacted semi-directly with the most MRFs (n = 5) in both screens. Without Cdk4/6 inhibition, *Ep300* was the node interacting with the greatest number of MRFs, including *Mef2a*, *Mef2c*, *Mef2d*, and *Myod*, whereas *Myod* was the MRF that interacted semi-directly with the most regulatory kinases (n = 8). These data point toward Src, Ep300 and MyoD as particularly important regulatory nodal points in the initial induction of Myogenin.

**Figure 4:**
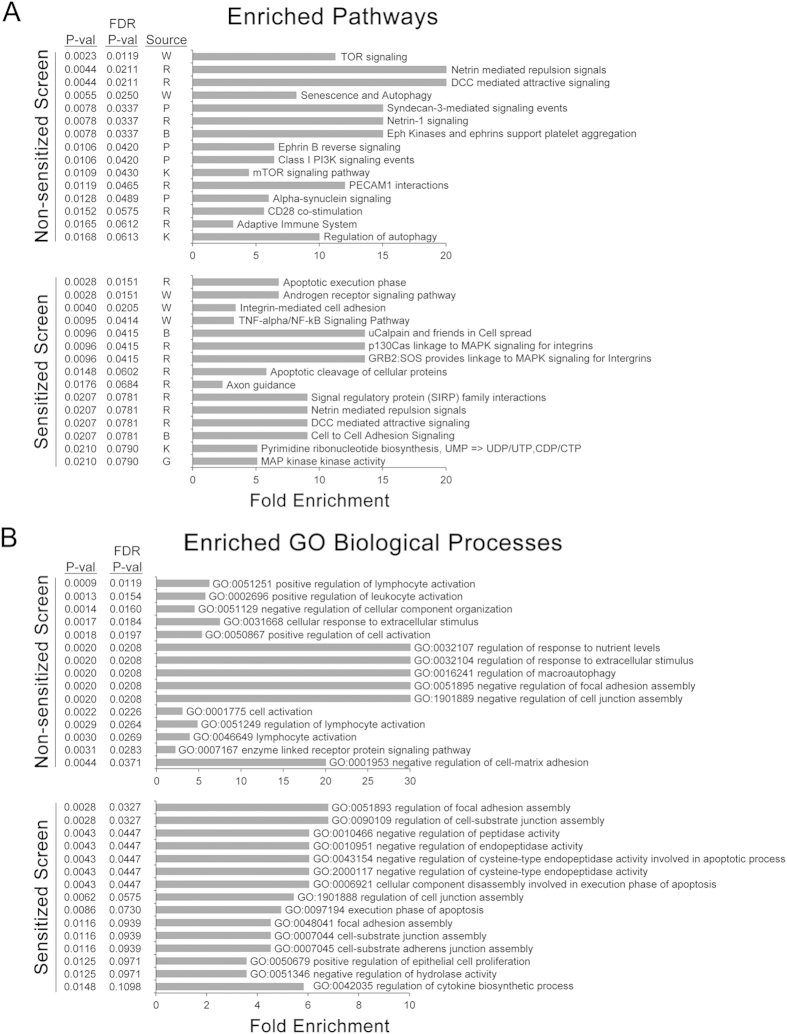
Pathway and gene ontology analysis identify processes linked with triggering skeletal muscle differentiation. Charts showing top 15 (**A**) biological pathways and (**B**) processes enriched with and without PD 0332991 as sensitizer. Fold enrichment is calculated as percentage of hits in the indicated pathway divided by the expected percentage based on the number of tested kinases in that pathway. Source indicates specific database: B, BioCarta; G, GenMAPP; K, KEGG; P, Pathway Interaction Database; R, Reactome; W, Wiki Pathways.

ToppGene^29^ analysis revealed a number of pathways that were enriched in the hit kinases, including mTOR, Eph/ephrins, and a variety of other receptor/kinase signaling pathways (Fig. 4A, top panel). As expected, including the Cdk4/6 inhibitor as a sensitizer revealed additional enriched pathways, including pyrimidine biosynthesis and Map kinase signaling (Fig. 4A, bottom panel). Many of the pathways identified in the sensitized and non-sensitized screen, and the processes identified by gene ontology analysis of hit kinases (Fig. 4B), highlight factors that directly or indirectly couple extracellular environmental cues to cytoskeletal changes and other intracellular signaling processes.

**Figure 5:**
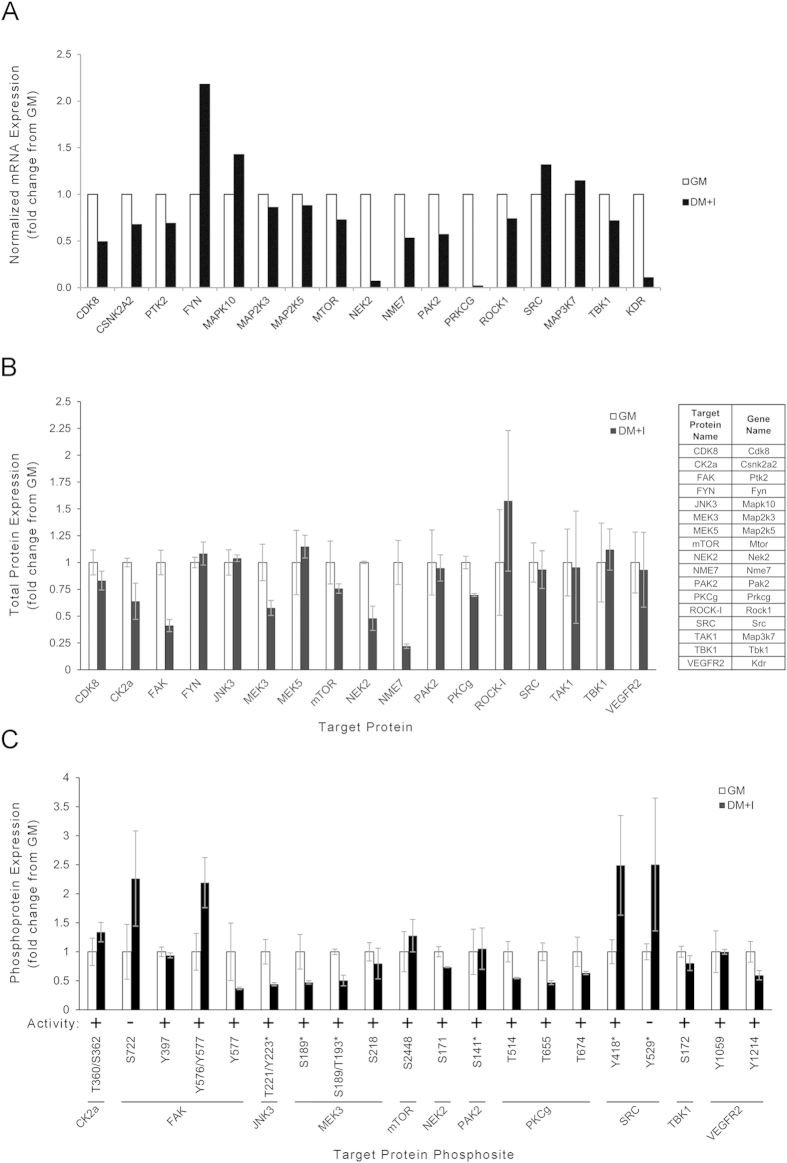
Decreased mRNA and protein/phosphoprotein expression of certain hit kinases correlates with muscle differentiation. (**A**) mRNA levels of a subset of the hits in growth medium (GM) versus differentiation medium (DM + I), determined by RNA-Seq. (**B**) Relative expression of protein (**B**) and specific phospho-amino acids (**C**) in a subset of the hits in GM versus DM + I, relative to HSC70 as a loading control, determined using KAM-880 protein microarray. Bars represent standard error for duplicate measurements. Phosphosite activity is listed as stimulatory ( + ) or inhibitory (−) of kinase activity when phosphorylated. Asterisks (*) indicate that an antibody has known or potential cross reactivity with corresponding phosphosite(s) on closely related protein(s). Note: In this figure, data are shown as relative to GM (values in GM are set as 1). The absolute expression of mRNA (**A**), expression of protein (**B**) and phospho-amino acid (**C**) relative to Hsc70 are shown in Supplementary Fig. S5.

We considered whether the hit kinases were differentially expressed in myoblasts versus differentiated myocytes. We employed RNA-Seq and a protein microarray to test the mRNA and protein expression and phosphorylation status of the hit kinases. We found that mRNA encoding many of the tested kinases was similar in GM and DM + I, but some decreased to varying degrees (Fig. 5A). In most of those cases, there was a corresponding decrease in the protein and activating phosphorylation sites (Fig. 5B,C). In the case of JNK3, encoded by Mapk10, neither mRNA nor protein decreased, but we found relative loss of phosphorylation of kinase-activating amino acid residues (Fig. 5C). We conclude that decreased expression or decreased phoshorylation in a subset of the hit kinases may represent a mechanism to couple decreased kinase activity with the initiation of myogenic differentiation.

### Secondary validation of mTOR and other kinases in myogenesis control

Many of the hits encode kinases that are well known negative regulators of muscle gene expression, such as Src^30^,^31^ and Rock1^32^,^33^. Consistent with previous studies, ectopic expression of Src repressed the activation of a plasmid reporter driven by the mouse *Ckm* promoter, and pharmacological inhibition of Src family kinases with dasatinib augmented the expression of muscle-specific genes in myoblasts maintained in GM (Supplementary Fig. S2). Some hits encode kinases that have not been well studied in myogenesis, such as Glyctk and Bub1b. We found that knockdown of *Glyctk* to about 60% of baseline significantly increased the mRNA expression of *Myog* and other muscle genes in C2C12 cells cultured in GM (Supplementary Fig. S3), revealing a role of *Glyctk* in repressing muscle differentiation genes.

**Figure 6:**
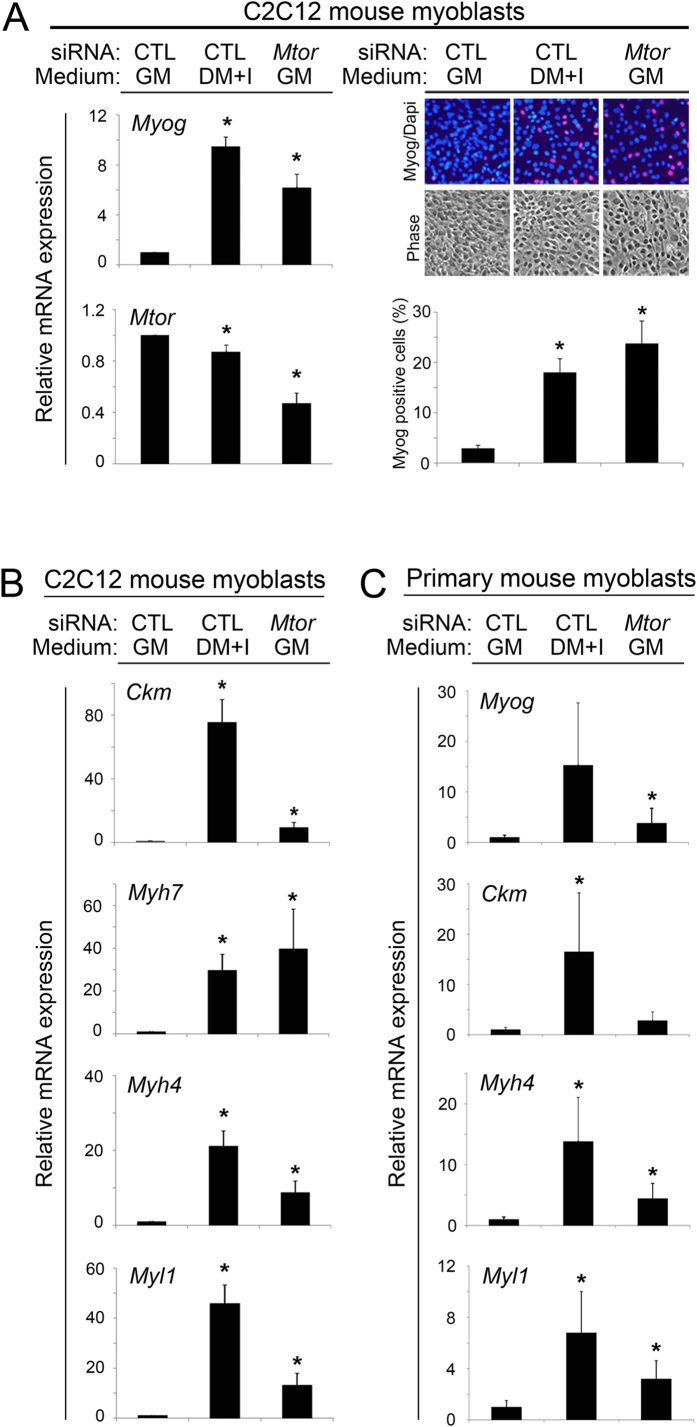
*Mtor* knockdown increases the expression of Myogenin and other muscle-specific differentiation genes in myoblasts. Charts showing mRNA or protein expression of indicated genes in (**A,B**) C2C12 cells and (**C**) primary mouse myoblasts cultivated in growth medium (GM) or differentiation medium (DM + I) with control (CTL) siRNA, *Mtor* knockdown, as indicated. In all cases, mRNA was quantified by qRT-PCR, normalized to *Gapdh*, and shown relative to expression in GM. Of note, *Myh7* was excluded from the primary myoblast studies (**C**) because it was not induced by DM + I. *p < 0.05 compared to GM; error bars represent standard deviation.

One of the hits, Mtor, caught our attention because mTOR signaling is widely viewed as a positive myogenesis regulator in cultured cells^18^,^33^,^34^,^35^,^36^. However, our screen identified it is a negative regulator. To confirm this role, we first tested whether transient knockdown of RNA encoding mTOR could increase *Myog* mRNA in cultured C2C12 myoblasts maintained in GM. Similar to the effects in the screen, *Mtor* knockdown increased *Myog* mRNA (Fig. 6A, left panels) and Myogenin protein (Fig. 6A, right panels). *Mtor* knockdown also increased the expression of several other muscle genes, and in some cases, the magnitude of induction approximated that achieved in cells cultivated in DM + I (Fig. 6B, left panels). In addition, we demonstrated that *Mtor* knockdown had similar effects in early-passage, mouse skeletal muscle satellite cells (Fig. 6C).

**Figure 7:**
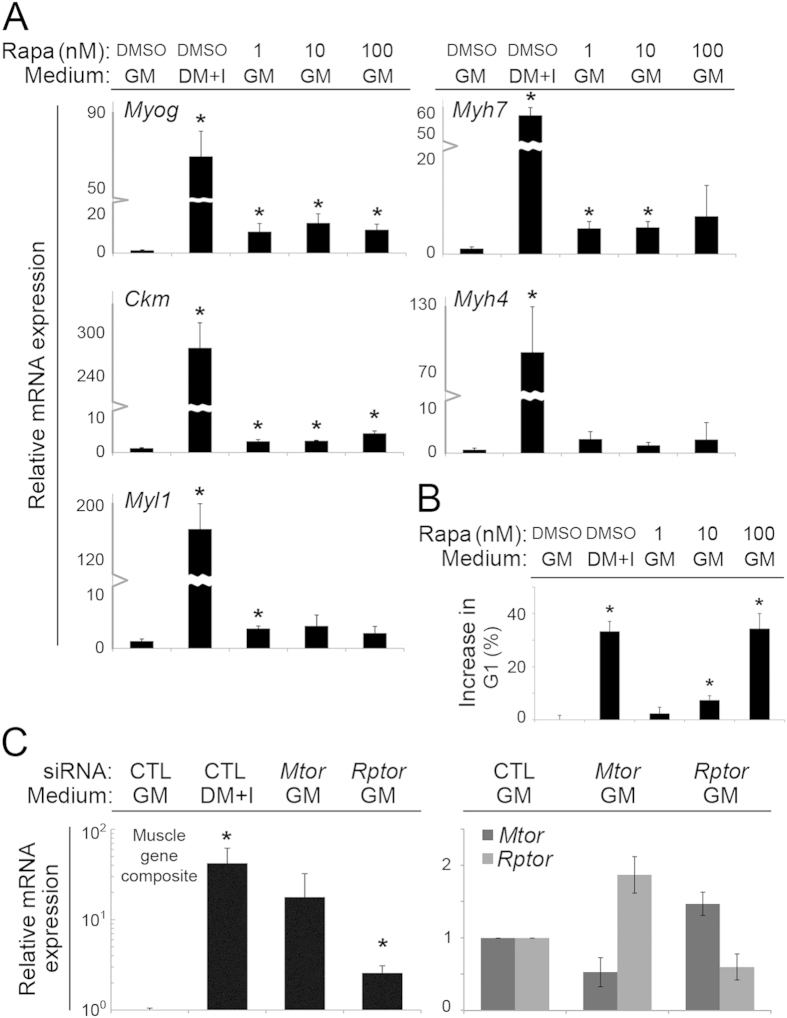
mTORC1 inhibition by rapamycin or *Rptor* knockdown increases muscle gene expression, independently from cell cycle arrest. (**A,B**) Quantification of (**A**) mature mRNA expression and (**B**) G_1_ cell cycle fraction in C2C12 myoblasts cultivated in growth medium (GM), differentiation medium with insulin (DM + I), or GM with rapamycin for 48 hours at the indicated doses (nM). (**C**) Aggregate quantification of muscle-specific signature genes (left) or indicated genes (right) in myoblasts cultivated as above with *Mtor* or *Rptor* siRNA knockdown. In all cases, mRNA quantified by qRT-PCR, normalized to *Gapdh*, and shown relative to expression in GM. CTL, scrambled siRNA control; *p < 0.05 compared to GM; error bars represent standard deviation.

We then tested whether chemical inhibition of mTOR could drive the initial steps in myogenic differentiation. The mTOR kinase sits at the heart of pathways that integrate environmental signals with intracellular processes guiding critical cell biological processes, positioning it well to guide the myoblast-to-myocyte transition. Although mTOR acts in two well-characterized complexes, we focused on the mTORC1 complex because it is closely coupled to the regulation of cell proliferation and protein translation^37^,^38^. Pharmacological inhibition of mTORC1 using rapamycin increased muscle gene expression in myoblasts maintained in GM and also arrested myoblasts in G_1_ phase of the cell cycle (Fig. 7A,B). Importantly, the magnitude of muscle gene induction did not correlate with the dose-dependent cell cycle arrest by rapamycin (Fig. 7B), indicating that its pro-differentiation effects were not merely related to its capacity to arrest proliferation.

The rapamycin findings suggested a specific role for Raptor in the mTORC1 complex. Interestingly, *Rptor* knockdown to approximately 60% of baseline only augmented expression of the five tested muscle genes by approximately 2.6-fold (range: 1.8 – 3.1) over baseline in GM, while parallel *Mtor* knockdown to ~53% increased their expression by ~18.8-fold (Fig. 7C). That the effects of rapamycin and *Rptor* knockdown are quantitatively smaller than *Mtor* knockdown suggests that *Mtor* knockdown likely does more to foster muscle gene expression than merely disrupt the mTORC1 complex. Further, mTORC1 is primarily thought to foster eIF-4E-dependent translation^39^,^40^. Our finding that knockdown of that translation factor was not sufficient to mimic the effects of *Mtor* or *Rptor* knockdown (Supplementary Fig. S4) suggests a role for additional mTOR effectors.

### mTOR inhibition increases transcription of MyoD-driven skeletal muscle promoters

**Figure 8:**
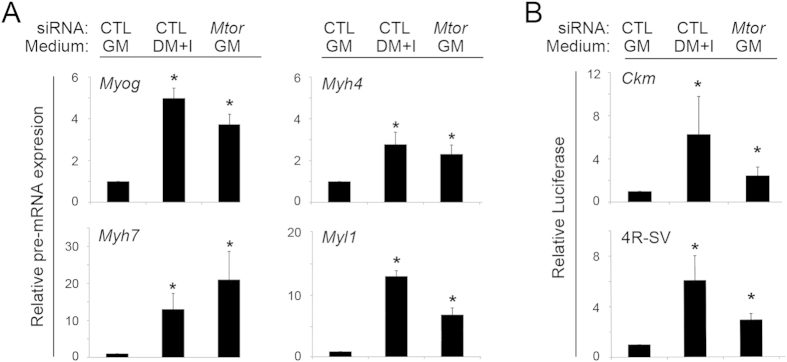
mTOR inhibition increases bHLH MRF-driven transcription of muscle-specific promoters. (**A**) Quantification of unprocessed RNA for four muscle-specific genes in C2C12 myoblasts cultivated in growth medium (GM) or differentiation medium (DM + I) and with control (CTL) siRNA or *Mtor* knockdown. mRNA was quantified by qRT-PCR, normalized to *Gapdh*, and shown relative to expression in GM. (**B**) Quantitation of luciferase in C2C12 cells cultivated as above, following transfection with reporter plasmids containing the Muscle Creatine Kinase promoter (*Ckm*) or a minimal SV40 promoter driven by four MyoD-specific “E” boxes (4 R-SV). Average values presented relative to baseline in GM. CTL, scrambled siRNA control; *p < 0.05 compared to GM; error bars represent standard deviation.

We investigated whether increased mRNA was due to increased transcription or post-transcriptional RNA stabilization. We first employed quantitative RT-PCR using primers specific for the nascent, unprocessed transcript. As was the case for the mature transcript, expression of the primary mRNA transcripts for the surveyed muscle genes increased when *Mtor* was knocked down (Fig. 8A). We also utilized transiently transfected plasmid reporters driven by the mouse *Ckm* promoter or a simplified reporter containing four, MyoD-specific, E-boxes (CANNTG) and a minimal promoter^16^; both of these reporters were induced in myoblasts cultivated in DM + I, and *Mtor* knockdown also increased the expression of both the complex and simplified promoters (Fig. 8B). Taken together, these data indicate that *Mtor* knockdown activates transcriptional programs driven by MyoD or related bHLH MRFs in myoblasts even though they are maintained in a mitogen-rich growth medium.

## Discussion

The molecular underpinnings of skeletal muscle lineage commitment and the consequent transcriptional induction of muscle specific genes are very well described^41^, but the factors that repress muscle differentiation genes in proliferating myoblasts are not as clear. Our findings provide new and largely unbiased insight into kinases that can accomplish this. As supported by our functional data, the inhibition of these individual kinases can represent a mechanism to initiate the muscle differentiation process, effectively guiding the transition between a proliferating, MyoD-expressing myoblast and a post-mitotic myocyte expressing Myogenin. Pathway and gene ontology analyses highlight the role that many of these kinases play to connect extracellular signals to intracellular events.

Many of our hits encode kinases known or suggested to play a role in skeletal muscle development. For example, the *Src* family kinases *Src* and *Fyn* were both identified in the non-sensitized screen. Ectopic expression of v-Src in quail and mouse myoblasts blocks the ability of myogenic bHLH proteins to induce Myogenin, whereas use of chemical inhibitors of Src or expression of a dominant negative form enhances Myogenin expression^30^,^31^. Pkn2 (also known as Prk2), identified in both sensitized and non-sensitized screens, can activate Fyn in keratinocytes^42^. The Rho kinase, Rock1, is known to negatively regulate later stages of myogenesis^32^,^33^, and has been implicated in signaling related to mTOR^33^, which we also identified as a negative regulator (see more below). Eph/ephrin signaling drives repulsive migration patterns in muscle satellite cells, but a direct role influencing muscle differentiation was not previously suspected^43^. Certain hit kinases also intersect with the aforementioned Wnt and Bmp signaling pathways, which positively and negatively influence muscle development, respectively^5^,^6^,^7^,^8^. For example, Bmp-dependent myogenesis suppression might be relieved by knockdown of *Acvrl1*, which encodes a Type I Tgfβ superfamily receptor^44^. Casein kinase I, encoded by *Csnk1a1*, can phosphorylate β-catenin and prime it for degradation in certain contexts^45^. *Cskn1a1* knockdown in myoblasts, then, would be expected to activate the canonical Wnt pathway, fostering muscle differentiation. Additional validation and mechanistic studies are needed to confirm these possible connections and extend them to *in vivo* settings.

We also identified mTOR as a negative regulator of muscle gene expression in myoblasts, and we confirmed that chemical inhibition of the mTORC1 complex is sufficient to foster the first steps in muscle differentiation. Positioning mTOR/mTORC1 activity as a negative regulator of the myoblast-to-myocyte transition is consistent with the role it plays to read extracellular cues from growth factors and nutrients that would be critical to a cellular “decision” to begin differentiation^34^. Our conclusion contrasts an elegant study from the Chen laboratory showing that *Mtor* knockdown decreases whereas *Rptor* knockdown increases muscle gene expression^46^. It is key to point out that their work focused on effects of knockdown in cells cultivated in differentiation medium, whereas our studies were performed in myoblasts maintained in GM. Ge and colleagues also demonstrate that Raptor governs phosphorylation of Serine-307 in IRS-1^46^, and phosphorylation of the orthologous site on human IRS-1 of leads to its degradation^47^. Rapamycin-dependent blockade of this phospho-serine in proliferating myoblasts would be predicted to increase IRS-1 to potentiate muscle gene expression.

It is important to note that mTOR signaling is also recognized as a positive myogenesis regulator in cultured cells^18^,^33^,^34^,^35^,^36^ and in experimental animals with muscle-specific targeting of *Mtor* and *Rptor*^48^,^49^. Hence, viewing our findings with these other reports highlights the biphasic role mTOR can play in myogenesis. In proliferating myoblasts, mTOR, and likely mTORC1 activity, helps to repress differentiation, whereas mTORC1 activation after differentiation facilitates the achievement of a terminally-differentiated state.

Mechanistically, mTOR/mTORC1 activity limits early muscle gene expression by blocking the capacity for myogenic bHLH proteins to augment the transcription of muscle specific promoters driven by reiterated MyoD-specific E-boxes. This is not likely to be a direct effect on MRFs because we were not able to identify the mTOR phosphorylation motif^38^ in human and mouse myogenic bHLH and Mef2 proteins (LX and SXS, unpublished data). The fact that eIF-4E knockdown does not mimic mTOR or Raptor knockdown speaks against a model in which mTORC1 acts by fostering eIF-4E-dependent protein translation^34^,^40^. Coupled with the fact that the observed pro-myogenic effects of rapamycin and *Rptor* silencing were relatively small when compared to *Mtor* knockdown, additional mechanistic studies should focus on other mTORC1 effectors as well as mTORC1-independent activities that may block MRF actions.

Finally, our results highlight the potential value of cell-based, high content screens to identify regulators of skeletal myogenesis. This type of approach has been previously leveraged using cultured myoblast lines^25^,^26^,^50^ and zebrafish embryos^27^ to study differentiation and lineage specification, respectively. Our screen differs from these prior efforts in two important ways. First, we have focused on identifying kinases that repress muscle differentiation in proliferating myoblasts. Second, we have focused on what could be construed as the initial step in the transition between a lineage-committed myoblast and one that has begun to differentiate: the induction of Myogenin. Elegant work from the Weintraub laboratory showed the transcriptional induction of Myogenin mRNA to be directly mediated by MyoD^28^, and this event is closely coupled to cell cycle arrest^51^,^52^. Others have successfully utilized myosin heavy chain, a later muscle differentiation marker^26^,^50^. Conceivably, that endpoint could be utilized in a pro-differentiation screen like ours to identify the subset of kinases that foster more complete differentiation when knocked down. A laudable goal for these types of screens could be to identify proteins whose activity might be manipulated therapeutically for skeletal myopathies or even to foster differentiation in rhabdomyosarcoma, a malignancy composed of myoblast-like cells. Given that multiple, forward-feeding signals are needed to achieve a terminally differentiated state, targeting multiple proteins is likely to be required.

## Methods

### Cell lines and reagents

Mouse skeletal myoblast C2C12 cells, purchased from American Tissue Culture Collection (Manassas, VA), were maintained as previously described^17^. FACS sorted mouse skeletal muscle satellite cells, obtained from Eric Olson (UT Southwestern Medical Center) and prepared as previously described^53^, were maintained with daily replenishment of medium consisting of Ham’s F-10 containing 20% FBS with 0.1 mg/ml Primocin (Invitrogen) and 5 ng/mL bFGF (Gibco) on Matrigel (BD Biosciences)-coated plates. To induce differentiation, myoblasts were cultivated in DMEM containing 2% horse serum (with 0.1 mg/ml Primocin for mouse muscle satellite cells) with or without 10 μg/mL insulin (Sigma Aldrich, St. Louis, MO) (DM or DM + I). All cell culture products were purchased from Gibco (Grand Island, NY) unless otherwise noted. Rapamycin and PD 0332991 (Selleckchem, Houston, TX) were resuspended in DMSO and used at concentrations as indicated.

### siRNA screen

The siRNA screen was carried out at the Quellos High Throughput Screening Core at the University of Washington, using the mouse kinase Silencer siRNA library (Ambion) targeting 571 kinases. It was arrayed onto two 384-well plates with 32 wells per plate designated for GM, 32 wells for DM + I, 32 or 37 wells for mock transfection, universal control, and *Kif11* positive knockdown control, and 288 or 283 wells for experimental transfection. Three different siRNA targeted against the same gene were pooled together in each well (17 nM final concentration for each siRNA, 50 nM final concentration total). Each condition, with and without sensitizer, was performed in triplicate.

C2C12 myoblasts were plated at 300 cells per well in 50 μL of DMEM with 10% FBS in poly-D-lysine coated 384-well plates (Corning, Corning, NY). Sixteen hours later, medium was removed, and the cells were transfected with Dharmafect 2 (Thermo Scientific, Lafayette, CO) in OptiMEM using a Biomek FX liquid handler. After 24 hours, transfection medium was replaced with GM in the experimental and negative control wells or DM + I in the positive control wells. Of note, half of the plates also received the sensitizer: PD 0332991 (1 μM) in 0.1% DMSO final concentration, which does not measurably promote differentiation in these cells^17^. Forty-eight hours later, the cells were fixed using paraformaldehyde (4% in PBS) prior to staining.

The fixed cells were stained using mouse anti-myogenin (F5D) antibody (BD Biosciences, San Jose, CA, Cat. No. 556358), which was detected using biotinylated anti-mouse antibody (Jackson ImmunoResearch, West Grove, PA, Cat. No. 715-065-150), Cy3-Streptavidin (Jackson ImmunoResearch, Cat. No. 016-160-084), and DAPI (Sigma Aldrich), essentially as described^17^. The stained plates were imaged using an ImageXpress Micro (Molecular Devices, Sunnyvale, CA) automated fluorescence microscope. Four sites were imaged per well using both DAPI and TRITC filters, and images were analyzed using MetaXpress 3.1 software (Molecular Devices).

### Secondary validation studies

Small interfering RNA (siRNA) used for secondary validation of *Mtor* was purchased from Ambion (Austin, TX). Non-targeting siRNA #3 (Thermo Scientific, Lafayette, CO) was used as a negative control. siRNAs (50 nM) were used with Dharmafect 2 to transfect 1.8 x 10^4^ C2C12 myoblasts/well seeded on 6-well plates. Sixteen hours after transfection, medium was changed to GM or DM + I, and cells were harvested for cell cycle analysis or qRT-PCR 48 hours later. The expression of five skeletal muscle mature mRNA transcripts (or four primary, unprocessed transcripts) was quantified by qRT-PCR using gene-specific primers (Supplementary Table S2) and stained with SYBR green, using the 7900HT Fast RealTime PCR System (Applied Biosystems), essentially as previously described^54^. Gene expression was normalized to *Gapdh* using the ΔΔCt method and compared to that in cells transfected with non-targeting siRNA.

### Protein and mRNA analyses

C2C12 cells were grown in GM or DM + I for 72 hours and harvested for either RNA-Seq or protein expression. RNA-Seq was carried out at the McDermott Next Generation Sequencing Core (UT Southwestern). Library preparation was performed using the TruSeq Stranded Total RNA LT Sample Prep Kit (Illumina, San Diego, CA), according manufacturer’s specifications. The library was quantified using a 2100 Bioanalyzer (Agilent Technologies, Santa Clara, CA) and the samples were sequenced on a HiSeq 2500 (Illumina) with 100 bp paired-end reads. RNA-Seq read quality was evaluated based on the Illumina purity filter and distribution of base quality scores at each cycle. Sequence reads for each sample were aligned to the hg19 version of the human reference genome assembly using Bowtie 2.1.0^55^ and the splicing-aware aligner TopHat 2.1.0^56^. The alignment allows only uniquely aligned reads and up to two mismatches per read. All other parameters were set to the default values. The quality of the RNA-Seq data was evaluated by FastQC (v0.10.1) and custom Perl (v5.16.1) and R (v3.0.1) scripts. Normalized gene expression level was calculated as fragments per kilobase per million fragments mapped (FPKM) by Cufflinks 2.0.2^57^ with default settings.

Expression of “hit” proteins or phosphorylation of specific residues on these proteins was accomplished using the Kinex KAM-880 Antibody Microarray (Kinexus Bioinformatics Corp., Vancouver, B.C.), according to manufacturer specifications. Briefly, the cells were lysed in Kinexus Lysis Buffer and protein concentration was measured by BCA assay (Thermo Scientific), 50 μg of total protein for each sample was dye-labeled, chemically cleaved using the Kinexus Chemical Cleavage Kit, and incubated on the microarray slide with the GM sample added to the control chamber and the DM + I sample added to the experimental chamber. The processed slide was returned to Kinexus for quantification and normalization. The corresponding proteins for hits from the siRNA screen were then identified and their expression values divided by the expression of HSC70, a loading control, to calculate fold change in GM and DM + I.

### Cell cycle and accumulation analyses

How chemical inhibition or knockdown of specific kinases influenced cell proliferation or accumulation was assessed using C2C12 myoblasts. Cell cycle analyses were performed using promidium iodide (PI) staining with cells fixed in 70% ethanol, processed as previously described^58^. Cell accumulation was assessed using CellTiter-Blue cell viability assay (Promega) with analysis performed using a POLARstar Omega microplate reacher.

### Muscle-specific promoter analyses

Plasmid DNA reporters, transiently transfected into C2C12 myoblasts using Lipofectamine (Invitrogen) according to manufacturer’s recommendations, were used to measure muscle specific promoter activity in cultured myoblasts. Plasmids included *p3300MCK-Luc*, containing 3300 bp from the muscle creatine kinase (*Ckm*) promoter, or *p4R-SV-Luc*, containing four MyoD-binding E-boxes and a minimal SV40 promoter – both analogous to previously described plasmids containing the chloramphenical acetyl transferase (CAT) reporter^12^,^16^ – as well as a control vector encoding *Renilla* luciferase driven by the thymidine kinase promoter (Promega). Transient transfection and luciferase assays were performed as previously described and expressed relative to internal control^54^.

### Gene ontology and pathway analysis

The lists of positively scoring kinase “hits”, with and without PD 0332991 as a sensitizer, were analyzed using ToppGene software^29^ ( https://toppgene.cchmc.org) to identify significantly enriched pathways and biological processes. A binomial test identified the pathways enriched in the hits as compared to their representation in the library; enriched pathways were identified if the probability of enrichment solely due to library content was less than 0.05. FDR corrected p-values were calculated using the Benjamini-Hochberg method.

The Human Protein Reference Database (HPRD) ( http://www.hprd.org) (accessed on 9/27/2012) and the Biological General Repository for Interaction Datasets (BioGRID) ( http://thebiogrid.org) (release 3.1.92) were utilized to explore potential interactions between hit kinases and certain myogenic regulators (MyoD, Myogenin, Myf5, Myf6, and Mef2A, C, and D). Maps showing the interactions were developed using Cytoscape^59^ as previously demonstrated^60^,^61^. Several hits – *Nek4*, *Epha10*, and *Peak1* without PD 0332991 and *Nek4*, *Peak1*, *Tk2*, and *Ror1* with PD 0332991 – displayed no connections in either database and were not included in the interaction maps.

### Additional statistical analyses

For the siRNA screen, data were normalized using the robust Z-score method^62^ and tested for significance using a *t*-test comparing the average percent Myogenin positive cells in three replicates for each siRNA target with that in the negative control (GM) wells on the same plates. An individual well scored positive if the knockdown caused a) the average percent Myogenin staining to increase significantly (*p* < 0.05 by Student’s *t*-test), and b) the average total number of cells in the triplicate wells to decrease by at least one median absolute deviation (MAD) from the controls. For cells receiving PD 0332991 as a sensitizer, total cell number was not included as a variable because this drug decreases myoblast cell number independently of siRNA knockdown^17^. “Hits” were only called if two of the replicates (or all three when the sensitizer was included) were positive.

For all other experiments, quantitative data were expressed as mean ± SD pooled from either multiple biological replicates from within one representative experiment or from multiple experiments, with statistical differences between two populations determined by Student’s *t*-test.

## Additional Information

**How to cite this article**: Wilson, R. A. *et al.* Negative regulation of initial steps in skeletal myogenesis by mTOR and other kinases. *Sci. Rep.* **6**, 20376; doi: 10.1038/srep20376 (2016).

## Supplementary Material

Supplementary Information
