# Supplementary material for: Negative regulation of initial steps in skeletal myogenesis by mTOR and other kinases
Source: Sci Rep. 2016 Feb 5;6:20376. doi: 10.1038/srep20376 (PMC4742887; doi:10.1038/srep20376)
Supplement: Supplementary Information [file srep20376-s1.pdf]

Supplementary Information

Negative regulation of initial steps in skeletal myogenesis by mTOR and other kinases

Raphael A. Wilson[1*], Jing Liu[1*], Lin Xu[1], James Annis[2], Sara Helmig[1], Gregory Moore[1], Casey Timmerman[1], Carla Grandori[2], Yanbin Zheng[1]#,Stephen X. Skapek[1]#

[1]Division of Hematology/Oncology, Department of Pediatrics, University of Texas Southwestern Medical Center, Dallas, TX; [2]Quellos High Throughput Screening Core, Department of Pharmacology, Institute for Stem Cell and Regenerative Medicine, University of Washington, Seattle, WA

[*]Contributed equally to this work

# To whom correspondence should be addressed: Yanbin Zheng, PhD, or Stephen X. Skapek, MD, Division of Hematology/Oncology, Department of Pediatrics, University of Texas Southwestern Medical Center, 5323 Harry Hines Blvd, MC 9063, Dallas, TX 75390; Telephone: 214-648-3081; FAX: 214-648-3122; Email: Yanbin.Zheng@utsouthwestern.edu or Stephen.Skapek@utsouthwestern.edu

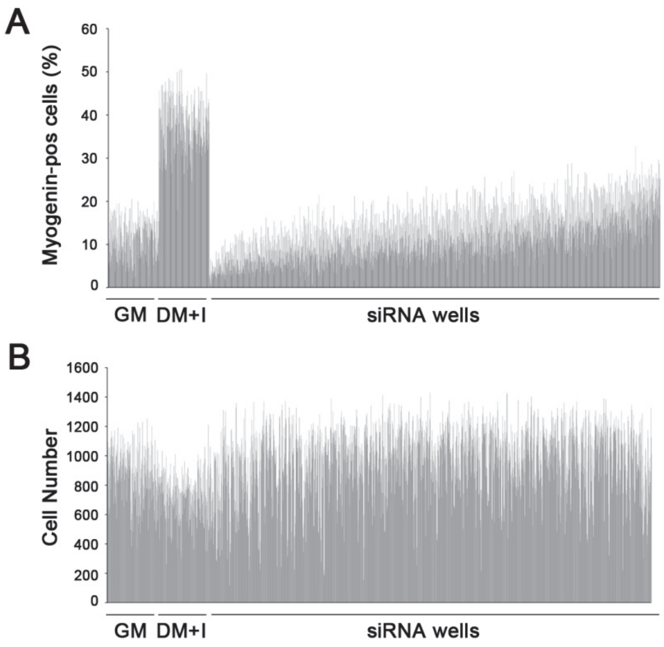

Supplementary Figure S1: Charts showing percent of cells expressing Myogenin (A) and total cell number (B) in myoblasts cultivated in the non-sensitized screen in GM, DM+I, or in GM plus siRNAs targeting individual kinases. GM, growth media; DM+I, differentiation media plus insulin.

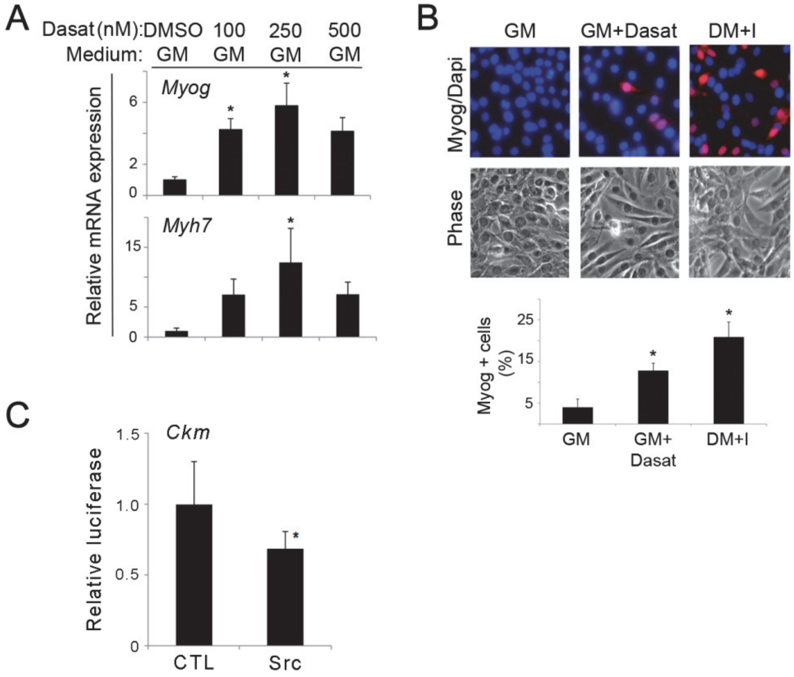

Supplementary Figure S2: <u>Src family kinase inhibition increases the expression of muscle-specific genes in mouse myoblasts and ectopic expression of mouse *Src* represses the activity of luciferase reporter driven by mouse *Ckm* promoter.</u> (A) Chart showing mRNA expression of indicated genes in C2C12 cells cultivated in GM following exposure to the Src kinase inhibitor dasatinib (Dasat) at 100, 250, or 500 nM concentration. In all cases, mRNA was quantified by qRT-PCR, normalized to *Gapdh*, and shown relative to expression in GM. (B) Representative photomicrograph (top) and quantification (bottom) of immunofluorescence staining for Myogenin (red) and Dapi (blue) in C2C12 cells cultivated in GM or DM+I and exposed to dasatinib (250 nM) or equal volume DMSO. In both (A) and (B), *, $p<0.05$ compared to GM; error bars represent standard deviation. (C) Charts showing quantitation of luciferase in C2C12 cells co-transfected with reporter plasmid containing the Muscle Creatine Kinase promoter (*Ckm*) and plasmids either empty (CTL) or containing mouse Src. Average

values presented relative to CTL. *, $p<0.05$ compared to CTL; error bars represent standard deviation.

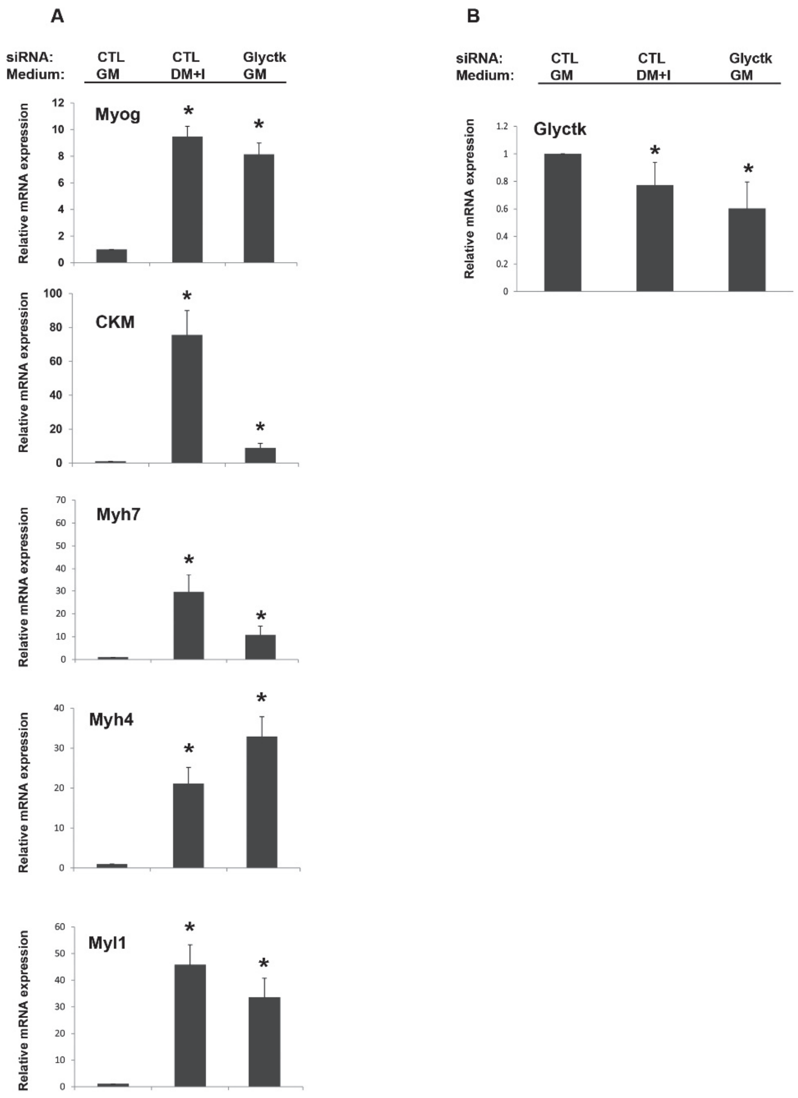

Supplementary Figure S3: <u>*Glyctk* knockdown increases the expression of multiple muscle-specific differentiation genes in myoblasts</u>. (A, B) Charts showing mRNA expression of indicated genes in C2C12 cells cultivated in growth medium (GM) or differentiation medium (DM+I) with control (CTL) siRNA, *Glyctk* knockdown, as indicated. In all cases, mRNA was quantified by qRT-PCR, normalized to *Gapdh*, and shown relative to expression in GM. *, $p<0.05$ compared to GM; error bars represent standard deviation.

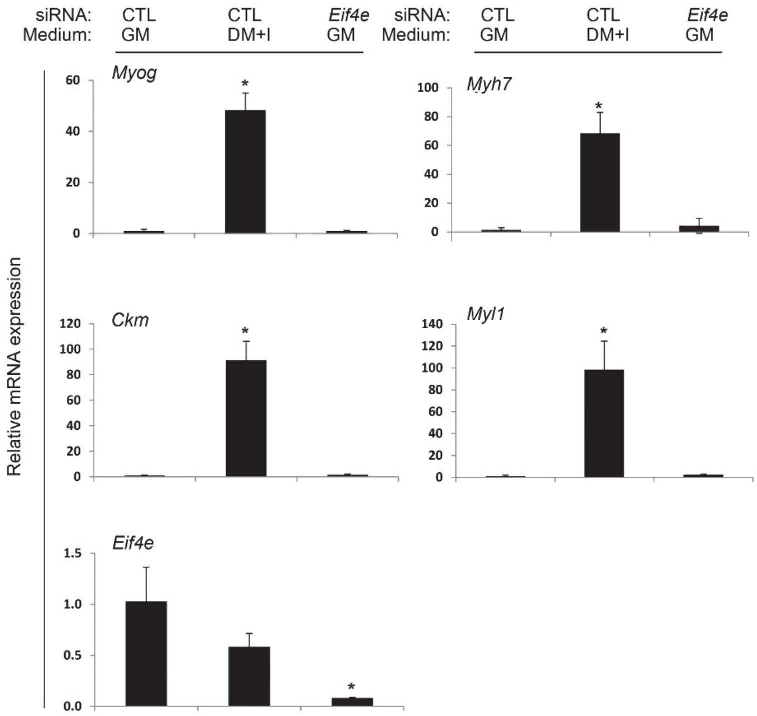

Supplementary Figure S4: <u>*Eif4e* knockdown has little effect on the expression of multiple muscle-specific differentiation genes in myoblasts</u>. Charts showing mRNA expression of indicated genes in C2C12 cells cultivated in growth medium (GM) or differentiation medium (DM+I) with control (CTL) siRNA, *Eif4e* knockdown, as indicated. In all cases, mRNA was quantified by qRT-PCR, normalized to *Gapdh*, and shown relative to expression in GM. *, $p<0.05$ compared to GM; error bars represent standard deviation.

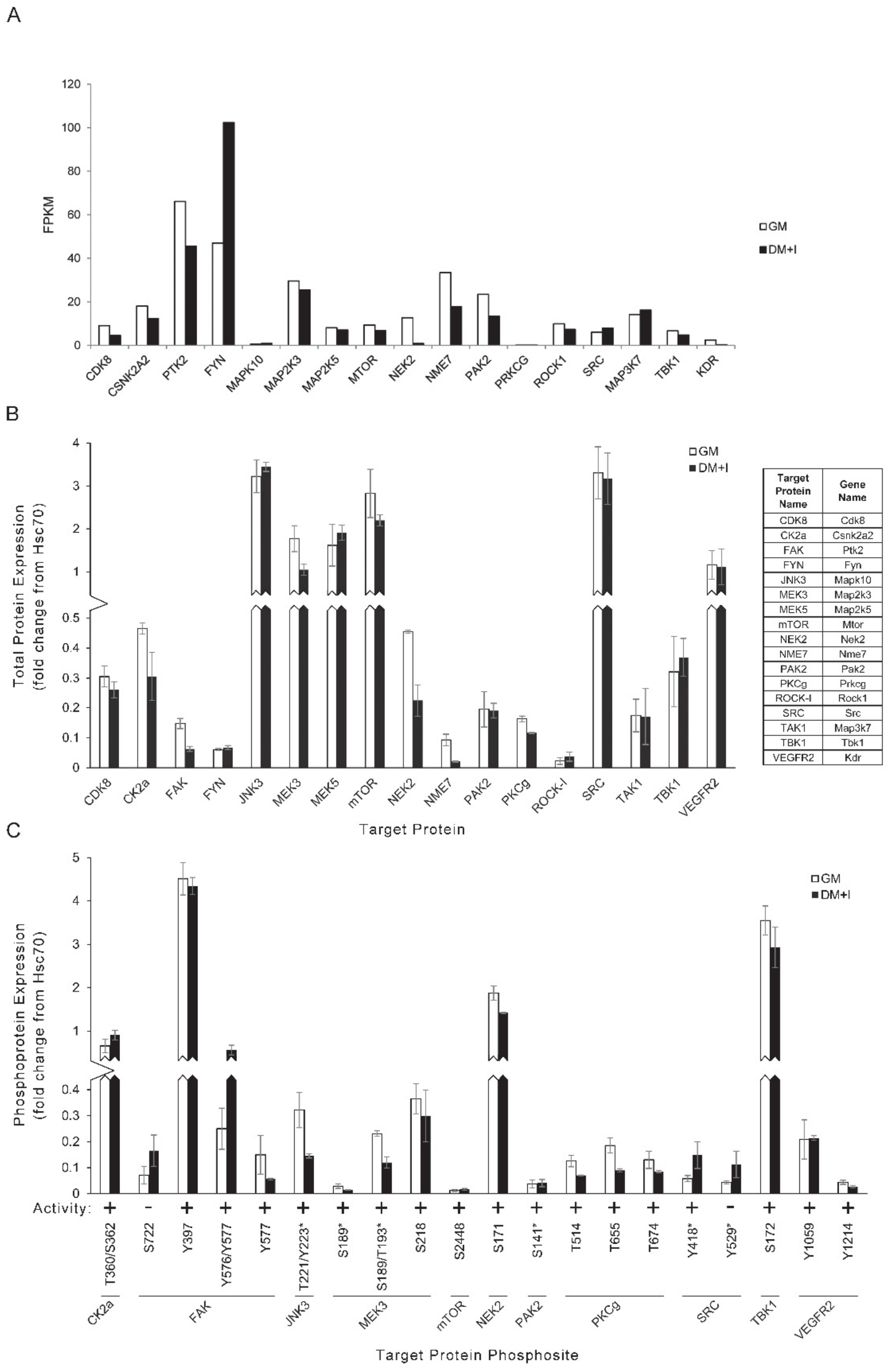

Supplementary Figure S5: Decreased mRNA and protein/phosphoprotein expression of certain hit kinases correlates with muscle differentiation. (A) mRNA levels of a subset of the hits in growth medium (GM) versus differentiation medium (DM+I), determined by RNA-Seq. (B) Relative expression of protein (B) and specific

phospho-amino acids (C) in a subset of the hits in GM versus DM+I, relative to HSC70 as a loading control, determined using KAM-880 protein microarray. Bars represent standard error for duplicate measurements. Phosphosite activity is listed as stimulatory (+) or inhibitory (-) of kinase activity when phosphorylated. Asterisks (*) indicate that an antibody has known or potential cross reactivity with corresponding phosphosite(s) on closely related protein(s).

A — Hits without PD-0332991

	Gene	Z-Score Mean for % Myogenin-positive	P-Value
	Nek2	3.520115826	2.27E-08
	Nek4	1.801030554	1.16E-07
	Src	2.572451612	1.47E-05
	Acvrl1	1.504573478	1.04E-04
	Prkcg	1.919450716	2.60E-04
	Axl	3.154742688	1.17E-02
•	Itk	1.143013201	1.18E-02
•	Fyn	0.934541538	1.25E-02
	Bub1b	2.479582197	1.34E-02
•	Mtor	1.484126693	1.36E-02
•	Ulk1	1.23933389	1.41E-02
	Pkn2	3.229870429	2.03E-02
	Gprk5	2.097037295	2.20E-02
•	Epha10	1.336331904	3.96E-02
•	Map3k7	1.358799005	4.22E-02
•	Prkaa2	1.422999458	4.24E-02
•	Epha4	1.495602843	4.42E-02
	Pip4k2b	2.622716068	4.70E-02
	Peak1	3.025273613	4.81E-02

B — Hits with PD-0332991

Gene	Z-Score Mean for % Myogenin-positive	P-Value
Cdk8	1.927397477	5.21E-23
Mpp6	3.757376863	3.09E-16
Nme2	1.747392336	3.90E-07
Tbk1	2.100182148	4.18E-07
Ror1	1.741779641	1.55E-05
Brsk2	1.47398229	2.47E-05
Glyctk	1.814575087	5.91E-04
Rps6ka3	2.301758758	6.78E-04
Nme7	1.627459259	1.04E-03
Pak2	3.459880586	1.11E-03
Prkaa1	1.880175247	3.51E-03
Stk39	1.777742053	4.85E-03
MapKapk3	4.789048154	4.88E-03
Mark2	3.33898968	6.02E-03
Mapk10	2.966408736	6.06E-03
Rock1	1.47316766	6.08E-03
Limk2	2.746004399	6.93E-03
Peak1	3.43575523	7.31E-03
Kdr	3.129823773	8.62E-03
Hipk3	2.388105972	8.87E-03
Mpp1	2.144455996	1.02E-02
Map2k3	2.139399942	1.05E-02
Nek4	3.386905134	1.09E-02
Ptk2	1.699562587	1.13E-02
Csnk1g2	1.930031442	1.26E-02
Axl	3.777125297	1.34E-02
Bckdk	1.7570191	1.61E-02
Nek1	2.29512273	1.90E-02
Pi4k2a	2.987622177	2.19E-02
Bmpr1a	2.551510537	2.19E-02
Pim1	2.503596988	2.24E-02
Nme4	3.059191095	2.51E-02
Pip4k2c	3.052988085	2.53E-02
Plxnb3	4.441856807	2.57E-02
Map2k5	3.420691704	3.08E-02
Csnk2a2	2.530345061	3.12E-02
Bub1b	2.551415687	3.23E-02
Csnk1a1	2.67146185	3.47E-02
Src	3.19427839	3.61E-02
Pkn2	3.215393677	3.65E-02
Mst4	1.974927741	4.33E-02
Tk2	2.36885744	4.49E-02

Supplementary Table S1: Listing of hit kinases, Z-score, and P value for the kinases identified as “hits” in the myogenic differentiation screen carried out without (A) and with (B) PD 0332991 included as a sensitizer.

qRT-PCR mRNA Primers

Gene	Sequence
Myog	F: ATCCAGTACATTGAGCGCCT
	R: GCTGTGGGAGTTGCATTCAC
Myh7	F: CTTGCTACCCTCAGGTGGCT
	R: GAGCCTTGGATTCTCAAACG
Ckm	F: CCTCCACAGCACAGACAGAC
	R: TGAGGTCTGGGTACTCCTCC
Myh4	F: GCAGGACTTGGTGGACAAAC
	R: AGCTCGTGCTGGATCTTACG
Myl1	F: AAAAATCGATCTGTCTGCTATTAAG
	R: GGTGATCTTGCATTCACCTGT
mTor	F: GCCAAAGCACTGCACTACAA
	R: CTCAGGCTGCTGGAGCTTAT
Glyctk	F: GACCCTTTGGAAGTGATTGCCAGT
	R: ACGGAGGCCATAGTGGTTGAGAAT

qRT-PCR Primary RNA Primers

Gene	Sequence
Myog 5'	F: CTACAGGCCTTGCTCAGCTC
	R: CCTATCCCTGGATCTGGTGT
Myog 3'	F: CAAGGGTCCCATACCAGTTG
	R: ACGATGGACGTAAGGGAGTG
Myh7 5'	F: AAGGGCATGAGGAAGAGTGA
	R: TGGCTAGGGAAAGGTAGCAA
Myh7 3'	F: ATGCTTGTGCCGTAGGAATG
	R: GCACATGCTCACACACTGC
Myh4 5	F: GAGAGGTTGGGAAGCATGAA
	R: TGTCGTACTTGGGAGGGTTC
Myh4 3	F: GCCACCAAATGACAGAACCT
	R: AGCTCGTGCTGGATCTTACG
Myl1 5'	F: TGTTTTAGCAAAGCCCAAGAA
	R: GCATCAAGAGATTAAAAGCATCA
Myl1 3'	F: CACAGCGTGCAAGTGTGATT
	R: GGCTTTGACACTCACGGTTC

Supplementary Table S2: Sequence of primers used for qRT-PCR amplification of mature and primary RNA transcripts for the indicated mouse genes.
